# Carbohydrate antigen 19-9 is a useful prognostic marker in esophagogastric junction adenocarcinoma

**DOI:** 10.1002/cam4.514

**Published:** 2015-08-26

**Authors:** Ryuma Tokunaga, Yu Imamura, Kenichi Nakamura, Tomoyuki Uchihara, Takatsugu Ishimoto, Shigeki Nakagawa, Masaaki Iwatsuki, Yoshifumi Baba, Yasuo Sakamoto, Yuji Miyamoto, Naoya Yoshida, Shinichiro Oyama, Takashi Shono, Hideaki Naoe, Hiroshi Saeki, Eiji Oki, Masayuki Watanabe, Yutaka Sasaki, Yoshihiko Maehara, Hideo Baba

**Affiliations:** 1 Department of Gastroenterological Surgery, Graduate School of Medical Sciences, Kumamoto UniversityKumamoto, Japan; 2 Department of Gastroenterology and Hepatology, Graduate School of Medical Sciences, Kumamoto UniversityKumamoto, Japan; 3 Department of Surgery and Sciences, Graduate School of Medical Sciences, Kyushu UniversityFukuoka, Japan; 4 Department of Gastroenterological Surgery, Japanese Foundation for Cancer ResearchTokyo, Japan

**Keywords:** Carbohydrate antigen 19-9, carcinoembryonic antigen, esophageal adenocarcinoma, esophagogastric junction, prognosisprognosis

## Abstract

The incidence rate of esophagogastric junction (EGJ) adenocarcinoma has been rapidly increasing worldwide. Carcinoembryonic antigen (CEA) and carbohydrate antigen 19-9 (CA19-9) are major serum tumor markers in gastrointestinal cancers. However, the role of these markers in EGJ adenocarcinoma has not been thoroughly investigated. A total of 211 patients with EGJ adenocarcinoma who underwent surgery or endoscopic submucosal dissection at two academic institutions, Kumamoto University Hospital or Kyushu University Hospital between January 1996 and March 2014, were eligible for this study. Serum CEA and CA19-9 were examined within 1 month before resection. The cut-off values for CEA and CA19-9 were set at 5.0 ng/mL and 37 U/mL, respectively. The clinicopathological features and prognostic roles of the markers were examined using univariate and multivariate analyses. The positive ratios for preoperative CEA (>5.0 ng/mL) and CA19-9 (>37 U/mL) were 20.3% and 12.9%, respectively. The positive ratio of CEA and CA19-9 was significantly higher in patients with tumors invading muscular or deeper layers (*P *=* *0.002 and <0.001, respectively). Cox proportional hazards model revealed that CA19-9 positivity, but not CEA positivity, was an independent prognostic factor in patients with EGJ adenocarcinoma for cancer-specific survival (multivariate hazard ratio [HR] = 3.89, 95% confidence interval [CI] 1.41–10.33; *P = *0.010) and overall survival (multivariate HR = 2.43, 95% CI 1.03–5.35; *P = *0.043). Preoperative serum CA19-9 is a useful prognostic marker in patients with EGJ adenocarcinoma.

## Introduction

Carcinoembryonic antigen (CEA) and carbohydrate antigen 19-9 (CA19-9) have been used as major serum tumor markers in various types of cancer for several decades. The glycoprotein CEA is widely used for assessing lung adenocarcinoma and stomach and colorectal cancers 1–3. CA19-9, a member of the Lewis family also known as sialyl Lewis A, is another general serum tumor marker used in gastrointestinal cancers, including pancreatic ductal adenocarcinoma (PDA) 4. Serum CEA level has been reported to correlate with disease stage and patient prognosis 5,6, and is the most sensitive marker for detecting recurrent disease 7 and monitoring the effects of chemotherapy during patient follow-up 4. CA19-9 plays a similar role in PDA 8,9.

The incidence rate of esophagogastric junction (EGJ) adenocarcinoma has been increasing rapidly in Western and Asian countries over the past few decades 10,11. According to the seventh edition of UICC classification, EGJ adenocarcinoma is included in esophageal adeonocarcinoma 12. An effective serum tumor marker would help to improve the clinical management of EGJ adenocarcinoma. However, few studies have examined the clinical utility of serum tumor markers in esophageal cancer including EGJ adenocarcinoma (Table1) 13–16.

**Table 1:** Previous studies reporting the association between preoperative serum tumor markers and outcomes in patients with esophageal cancer

Reference	Year of publication	No. patients (No. ESCC/No. EAC)	CEA cut-off value (ng/mL)	CEA-positive ratio (%)	CA19-9 cut-off value (U/mL)	CA19-9 positive ratio (%)	SCC antigen cut-off value (ng/mL)	SCC antigen positive ratio (%)	*P-*value for overall survival					
									Univariate analysis			Multivariate analysis		
									CEA	CA19-9	SCC	CEA	CA19-9	SCC
Clark et al. 13	1995	100 (20/80)	5.0	19.0	—	—	—	—	0.690	—	—	—	—	—
Mroczko et al. 14	2008	89 (63/26)	4.0	17.0	—	—	2.0	64.0	0.488	—	0.468	—	—	—
Lukaszewicz-Zajac et al. 15	2012	53 (30/23)	4.0	30.0	—	—	2.0	24.0	0.728	—	0.127	—	—	—
Scarpa et al. 16	2014	243 (82/161)	5.0	14.7	37.0	12.3	—	—	—	—	—	—	—	—

“—” means that there is no examination. ESCC, esophageal squamous cell carcinoma; EAC, esophageal adenocarcinoma; CEA, carcinoembryonic antigen; CA19-9, carbohydrate antigen 19-9; SCC, squamous cell carcinoma.

The aim of this study was to investigate the clinical usefulness of serum CEA and CA19-9 in patients with EGJ adenocarcinoma, utilizing a dataset from two academic institutions. We also hypothesized that CEA or CA19-9 can be a prognostic marker in EGJ adenocarcinoma.

## Material and Methods

### Patients

Consecutive 220 patients with EGJ adenocarcinoma who were treated at Kumamoto University Hospital (Kumamoto, Japan) between January 1996 and March 2014, or at Kyushu University Hospital (Fukuoka, Japan) between April 2005 and March 2014. Survival data were available in all cases. Among them, nine cases, for which neither CEA nor CA19-9 data were available, were excluded. Exclusion criteria were shown in Figure S1. Finally, a total of 211 patients were enrolled in this study. There were 167 (79.1%) men and 44 (20.9%) women. The mean age of the patients was 67.8 years (range 33–89 years). Surgical resection was performed in 196 (92.9%) patients at the Department of Gastroenterological Surgery, Kumamoto University Hospital or Kyushu University Hospital, and endoscopic submucosal dissection (ESD) was performed in 15 (7.1%) at the Department of Gastroenterology, Kumamoto University Hospital. Of the 196 surgically resected cases, 181 (92.3%) cases underwent surgery with curative intent and 15 (7.7%) cases with palliative intent.

### Study design

Treatment dates were obtained retrospectively from patients’ records. Cases were observed until patients’ death or 28 February 2015, whichever came first. The mean follow-up time was 34.1 months (range, 1–118 months). Disease staging was performed according to the seventh edition of UICC classification. Use of the clinical data was approved by the Human Ethics Review Committee of the Graduate School of Medicine, Kumamoto University and Kyushu University.

### Tumor markers

Serum CEA and CA19-9 were tested within 1 month prior to surgery or ESD according to the manufacturer’s instructions. The cut-off values for serum CEA and CA19-9 were set at 5.0 ng/mL and 37 U/mL based on previous reports, respectively 13,16.

### Statistical analysis

All statistical analyses were performed using JMP statistical software package version 10 (SAS Institute Inc., Cary, NC) and Excel 2010 (Microsoft, Redmond, WA). Univariate analyses were performed to investigate clinicopathological factors and tumor markers. Chi-square test (case number ≥5) or Fisher’s exact test (case number <5) was used for categorical data (sex, tumor location by Siewert classification, depth of tumor invasion, lymph node metastasis, distant metastasis, histopathological types, and treatment method), and Student’s *t*-test was used for age. The *P*-value for significance was adjusted by Bonferroni correction to *P *=* *0.0063 (=0.05/8) to account for multiple hypothesis testing in associations between tumor markers and the other eight covariates.

Survival analysis was performed in patients for whom both CEA and CA19-9 data were available (*n* = 188; survival analysis dataset, Fig. S1). The Kaplan–Meier method and log-rank test were used for survival analysis according to the tumor marker status (CEA ≤5.0 vs. >5.0 ng/mL and CA19-9 ≤ 37 vs. >37 U/mL). For analyses of cancer-specific mortality, deaths as a result of other causes were censored. Cox proportional hazards regression models were used to calculate hazard ratios (HRs) and 95% confidence intervals (CIs) according to the tumor marker status. The proportionality of hazard for CEA or CA19-9 was confirmed by the graph of the log[−log(survival probability)] versus log of survival time graph. A multivariate model initially included the following clinicopathological variables: age (<68 vs. ≥68 years; divided into two groups by median age of 68 years), sex, tumor location (Siewert type I–II vs. III), depth of tumor invasion (mucosal or submucosal layer vs. muscular or deeper layer), lymph node metastasis (negative vs. positive), distant metastasis (negative vs. positive), histopathological types (well-moderate vs. poorly), and treatment method (ESD vs. surgery). Backward elimination was performed with a threshold of *P *=* *0.05 to avoid overfitting. The *P*-value for significance was adjusted by Bonferroni correction to *P *=* *0.0125 (=0.05/4) to account for a hypothesis testing in associations between two tumor markers (CEA and CA19-9) and two survival analyses (cancer-specific and overall survival).

## Results

### Correlations between serum tumor markers positivities and clinicopathological factors

Among the total of 220 patients, preoperative serum CEA was examined in 197 (93.4%) and CA19-9 in 202 (95.7%). The median serum CEA and CA19-9 antigen concentrations were 2.3 ng/mL (0.1–105.2 ng/mL) and 10.1 U/mL (0.1–7440 U/mL), respectively. Serum CEA positivity (>5.0 ng/mL) was observed in 40 (20.3%) cases and CA19-9 positivity (>37 U/mL) in 26 (12.9%) cases. A significant correlation was observed between CEA and CA19-9 positivities (*P *<* *0.001) (Fig. S2). The positive ratios of CEA and CA19-9 were significantly higher in patients with tumors invading muscular or deeper layers (*P *=* *0.002 and <0.001, respectively) (*P *≤* *0.0063 [=0.05/8] after Bonferroni correction). Besides, the lymph node metastasis showed borderline significant associations with CEA positivity and CA19-9 positivity (*P *=* *0.010 and 0.022, respectively) (Table2).

**Table 2:** Associations between CEA and CA19-9 positivities and clinicopathological factors in patients with EGJ adenocarcinoma

	CEA		*P-*value	CA19-9		*P-*value
	Negative	Positive		Negative	Positive	
No. patients (%)	157 (80)	40 (20)		176 (87)	26 (13)	
Age			0.270			0.163
Mean ± SD	67.2 ± 11.7	69.4 ± 10.5		67.3 ± 11.7	70.7 ± 8.8	
Sex (%)			0.151			0.782
Male	122 (78)	35 (88)		138 (78)	21 (81)	
Female	35 (22)	5 (12)		38 (22)	5 (19)	
Siewert classification (%)			0.269			0.186
I	12 (8)	2 (5)		14 (8)	4 (15)	
II	83 (63)	27 (68)		94 (53)	16 (62)	
III	62 (39)	11 (27)		68 (38)	6 (23)	
Depth of tumor invasion (%)			0.002			<0.001
T1	78 (50)	9 (22)		87 (49)	3 (12)	
T2	20 (13)	3 (8)		19 (11)	3 (12)	
T3	43 (27)	19 (47)		51 (29)	12 (46)	
T4	16 (10)	9 (23)		19 (11)	8 (30)	
Lymph node metastasis (%)			0.010			0.022
Negative	106 (68)	18 (45)		116 (66)	11 (42)	
Positive	51 (32)	22 (55)		60 (34)	15 (58)	
Distant metastasis (%)			0.164			0.440
Negative	148 (94)	35 (88)		163 (93)	23 (88)	
Positive	9 (6)	5 (12)		13 (7)	3 (12)	
Histopathological types (%)			0.822			0.782
Well-moderate	111 (71)	29 (73)		124 (70)	19 (73)	
Poorly	46 (29)	11 (27)		52 (30)	7 (27)	
Treatment method (%)			0.976			0.05
ESD	12 (7)	3 (8)		13 (7)	0 (0)	
Surgery	145 (93)	37 (92)		163 (93)	26 (100)	

(%) indicates the proportion of cases with a specific clinicopathological factor among each CEA or CA19-9 status group. The *P-*value for significance was adjusted for multiple hypothesis testing to *P *=* *0.05/8 = 0.0063. Thus, a *P-*value between 0.05 and 0.0063 should be regarded as of borderline significance. CEA, carcinoembryonic antigen; CA19-9, carbohydrate antigen 19-9; EGJ, esophagogastric junction; ESD, endoscopic submucosal dissection.

**Figure 1:**
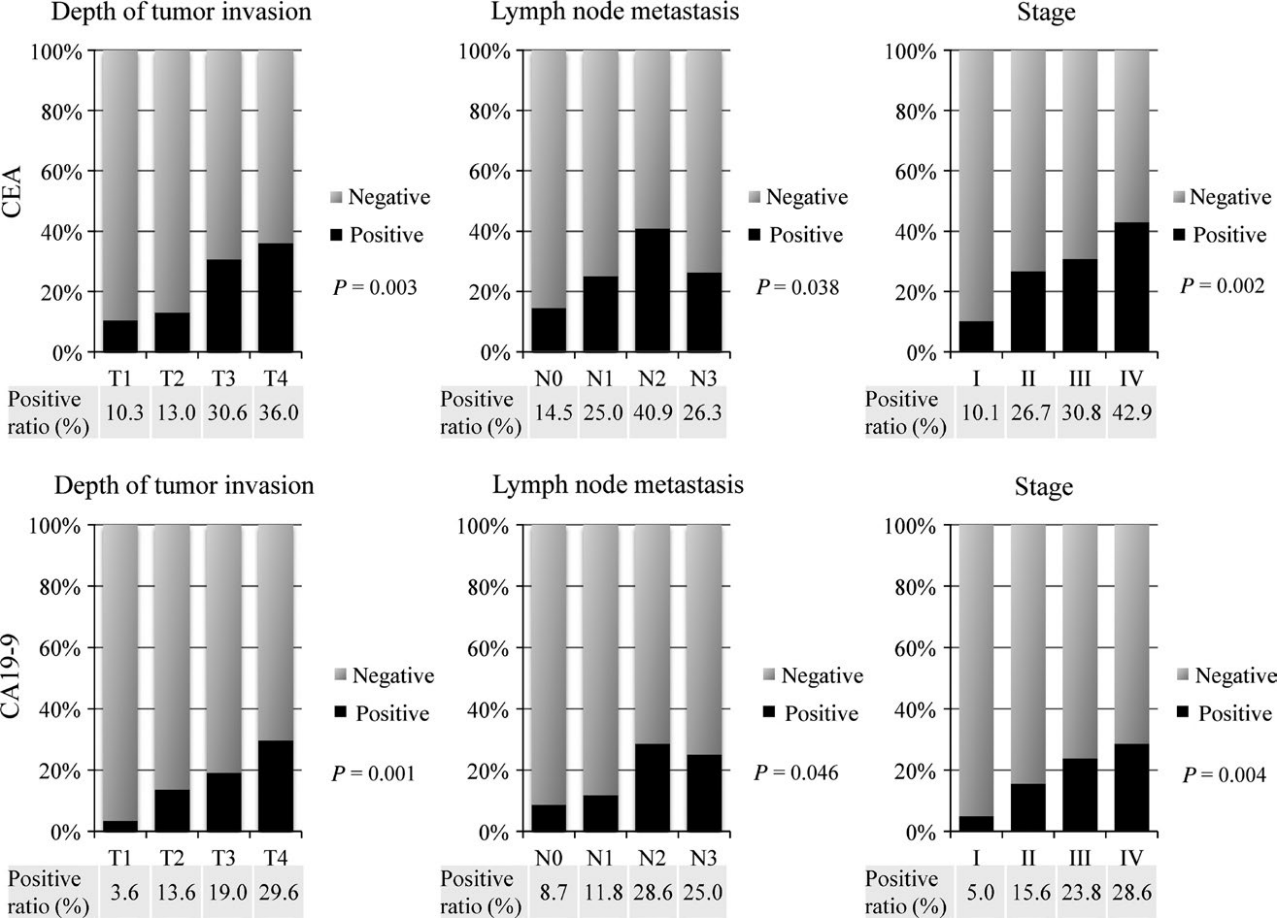
Incidences of CEA and CA19-9 positivities according to the depth of tumor invasion, lymph node metastasis, and stage of EGJ adenocarcinoma. CEA, carcinoembryonic antigen; CA19-9, carbohydrate antigen 19-9; EGJ, esophagogastric junction.

The detailed distributions of CEA and CA19-9 according to the depth of tumor invasion, lymph node metastasis, and disease stage are shown in Figure1. Positive ratios for both CEA and CA19-9 also increased with advancing stage.

### Correlations between serum tumor markers positivities and patient outcomes

**Figure 2:**
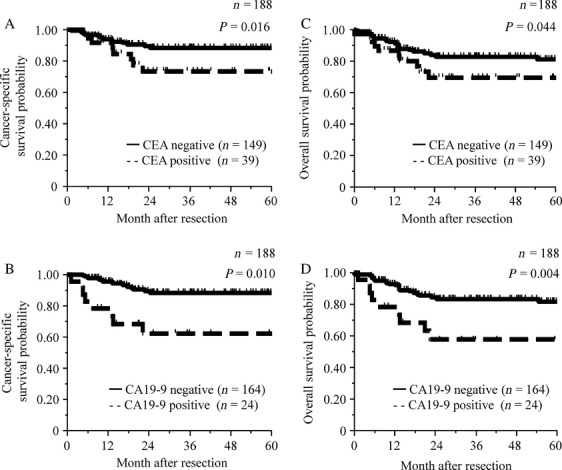
Cancer-specific survival curves according to the preoperative CEA positivity (A) and CA19-9 positivity (B). Overall survival curves according to the preoperative CEA positivity (C) and CA19-9 positivity (D). CEA, carcinoembryonic antigen; CA19-9, carbohydrate antigen 19-9.

The 5-year cancer-specific survival probabilities were 73.3% for CEA-positive (>5.0 ng/mL) and 88.3% for CEA-negative (≤5.0 ng/mL) cases, and 62.4% for CA19-9-positive (>37 U/mL) and 88.6% for CA19-9-negative (≤37 U/mL) cases in the survival analysis dataset (*n *=* *188). In addition, the 5-year overall survival probabilities were 69.4% for CEA-positive (>5.0 ng/mL) and 81.1% for CEA-negative (≤5.0 ng/mL) cases, and 57.6% for CA19-9-positive (>37 U/mL) and 81.7% for CA19-9-negative (≤37 U/mL) cases. In log-rank test, CEA (>5.0 ng/mL) and CA19-9 (>37 U/mL) positivity were significantly associated with poorer cancer-specific survivals (*P *=* *0.016 and 0.010, respectively) and overall survivals (*P *=* *0.044 and 0.004, respectively) (Fig.2). However, Cox multivariate analysis importantly showed that CA19-9 positivity (>37 U/mL), but not CEA (>5.0 ng/mL) positivity, was an exclusive independent prognostic factor for cancer-specific survival (multivariate HR = 3.89, 95% CI 1.41–10.33; *P *=* *0.010) and overall survival (multivariate HR = 2.43, 95% CI 1.03–5.35; *P *=* *0.043) (Table3).

**Table 3:** Univariate and multivariate^1^ analysis for cancer-specific survival and overall survival in the patients with EGJ adenocarcinoma (*n* = 188)

	No. patients (%)Positive/negative	No. events (%)Events/*n*	Univariate analysis			Multivariate analysis		
			HR	95% CI	*P-*value	HR	95% CI	*P-*value
Cancer-specific survival								
CEA	39 (21)/149 (79)	23 (12)/188	2.70	1.12–6.16	0.028	1.33	0.53–3.15	0.533
CA19-9	24 (13)/164 (87)	23 (12)/188	4.30	1.73–9.93	0.003	3.89	1.41–10.33	0.010
Overall survival								
CEA	39 (21)/149 (79)	34 (18)/188	2.07	0.97–4.17	0.060	—2	—2	2
CA19-9	24 (13)/164 (87)	34 (18)/188	2.90	1.28–6.01	0.013	2.43	1.03–5.35	0.043

The *P-*value for significance was adjusted to *P *=* *0.05/4 = 0.0125. Thus, a *P-*value between 0.05 and 0.0125 should be regarded as of borderline significance. EGJ esophagogastric junction; HR, hazard ratio; CEA, carcinoembryonic antigen; CA19-9, carbohydrate antigen 19-9.

1 Adjusted for age, sex, tumor location by Siewert classification, depth of tumor invasion, lymph node metastasis, distant metastasis, histopathological types, and treatment method.

2 CEA variable was not included in the final model of multivariate analysis because of backward elimination (*P *>* *0.05).

As CA19-9 was significantly associated with tumor depth of invasion, the survival probabilities according to the CA19-9 status were also examined in various subgroups of T stages (T1, T2–3, or T4, Fig. S3). Intriguingly, CA19-9 had a greater prognostic impact in the patients with T4 tumor, compared to those with T1, or T2–3 tumor. In addition, survival probabilities were examined in combination of CEA and CA19-9 positivity status. Of note, CEA-negative/CA19-9-positive cases (*n* = 12) experienced worst prognosis (5-year survival rate, 50.0%), in both cancer-specific and overall survival (Fig. S4).

## Discussion

The current results represent the largest study to examine the prognostic roles of CEA and CA19-9 in patients with EGJ adenocarcinoma, utilizing a dataset from two academic institutions. This study revealed an incidence of CA19-9 positivity (>37 U/mL) of 12.9% in EGJ adenocarcinoma, which was significantly correlated with depth of tumor invasion and was an independent prognostic factor.

Few studies have investigated the clinical utility of serum tumor markers in EGJ adenocarcinoma 13–16, and these reports combined the results of patients with esophageal squamous cell carcinoma and esophageal adenocarcinoma. In lung cancer, CEA is a sensitive marker for adenocarcinoma, while squamous cell carcinoma antigen and cytokeratin 19 fragment are markers for squamous cell carcinoma, and neuron-specific enolase and progastrin-releasing peptide are markers for small cell carcinoma 17,18. Useful tumor markers may thus differ between squamous cell carcinoma and adenocarcinoma. The current study was the first to assess the prognostic usefulness of tumor markers in a single histological type of EGJ adenocarcinoma.

In a recent report, Scarpa et al. demonstrated the usefulness of preoperative serum CEA and CA19-9 levels for detecting occult advanced esophageal adenocarcinoma 16. In their study, the incidence of CA19-9 positivity was 12.3%, which was similar to that found in our study. Although they did not examine the prognostic role of CA19-9, they showed that preoperative positivity for CA19-9 had higher sensitivity and specificity for advanced stage compared with CEA, which also supports our conclusion that CA19-9 is a more useful prognostic marker than CEA. Our study also showed that CA19-9 positivity was significantly correlated with depth of tumor invasion. One possible mechanism to explain this observation is that tumor cells in locally advanced cases are surrounded by a hypoxic environment, which induces CA19-9 19. Of note, our study showed that CA19-9 is a prognostic marker, beyond tumor depth. EGJ adenocarcinoma is associated with inflammation of gastroesophageal reflux disease, and inflammation drives cancer progression 20,21. CA19-9 is also known as a marker of inflammation 22. Our data suggested that CA19-9 positivity may represent an inflammation, which may display more aggressive phenotype rather than tumor depth of invasion, in the patient with EGJ adenocarcinoma.

In this study, the positive ratio of preoperative CA19-9 (12.9%) was relatively small. However, for instance, in colorectal cancer, *BRAF* mutations status is an important and useful prognostic marker 23, despite its low prevalence rate of around 5%. Considering that CA19-9 positivity possessed a significant prognostic impact on EGJ adenocarcinoma in this study, the proportion of 12.9% was not ignorable, but may be useful in the management of this cancer, such as in decision of neoadjuvant treatment application.

Attempts have been made to predict the prognosis in patients with EGJ adenocarcinoma. Human epidermal growth factor receptor 2 (HER-2), cyclooxygenase 2 (COX2) and urokinase-type plasminogen activator receptor (uPAR) have all been reported to be prognostic markers in EGJ adenocarcinoma. HER2, a member of the epidermal growth factor receptor family, is an important target of molecular therapy for breast and gastric cancers 24,25. Although some studies found a prognostic relevance of HER2 amplification in EGJ adenocarcinoma 26,27, Okines et al. found no such association 28, and the clinical utility of HER2 status in EGJ adenocarcinoma thus remains controversial. COX2, a component enzyme of the arachidonic acid cascade, is known to correlate with inflammation and cancer proliferation 29. High COX2 expression in EGJ adenocarcinoma identified by immunohistochemistry (IHC) has been reportedly associated with lymph node metastases and poor outcome 30,31. uPAR, part of the plasminogen activation system, is known to be highly expressed in various malignant tumors 32. Laerum et al. reported that tumors positive for uPAR by IHC showed more malignant behavior and were associated with shorter survival compared with uPAR-negative tumors 33. However, although these molecular markers may be potentially useful, they need to be tested in resected specimens and are therefore not applicable in preoperative settings or unresectable patients. In contrast, CA19-9 testing is available irrespective of patient disease status, whenever a blood test can be performed, and is a commonly used serum tumor marker in gastrointestinal cancers worldwide.

There were some limitations to this study. First, the data of chemotherapeutic use were not available in this study. Nonetheless, treatment decision making was based on TNM stage in this study, and it seems unlikely that chemotherapy use differed substantially by CA19-9 status. Second, relapse-free survival was not available in this study. However, cancer-specific survival is a reasonable surrogate of esophageal cancer-specific outcome. Finally, our dataset was collected retrospectively. Therefore, we could not exclude the patients with comorbid inflammatory diseases, or those without Lewis antigen, which can affect the positivity of CA19-9. CA19-9 has occasionally been shown to be increased in inflammatory conditions 22, and the patients without Lewis antigen (5–10% population) are unable to produce CA19-9 34,35. Larger scale prospective studies accounting for these factors are needed to confirm our findings.

In conclusion, this study demonstrated that CA19-9 may be a useful prognostic serum tumor marker in patients with EGJ adenocarcinoma. Additional, large-scale studies are warranted to assess the clinical utility of CA19-9 testing in EGJ adenocarcinoma.

## Conflict of Interest

None declared.

## Supporting Information

**Figure S1.** Flow diagram for included cases for this study. CEA, carcinoembryonic antigen; CA19-9, carbohydrate antigen 19-9.

**Figure S2.** Associations between preoperative serum CEA and CA19-9. CEA, carcinoembryonic antigen; CA19-9, carbohydrate antigen 19-9.

**Figure S3.** (A, C, and E): Cancer-specific survival curves according to the preoperative CA19-9 positivity in T1, T2–3, or T4 tumors. (B, D, and F): Overall survival curves according to the preoperative CA19-9 positivity in T1, T2–3, or T4 tumors. CA19-9, carbohydrate antigen 19-9.

**Figure S4.** (A) Cancer-specific and (B) overall survival curves in combination of CEA and CA19-9 positivity status. CEA, carcinoembryonic antigen; CA19-9, carbohydrate antigen 19-9.
