# Supplementary figures and images for: Carbohydrate antigen 19-9 is a useful prognostic marker in esophagogastric junction adenocarcinoma
Source: Cancer Med. 2015 Aug 26;4(11):1659–66. doi: 10.1002/cam4.514 (PMC4673992; doi:10.1002/cam4.514)

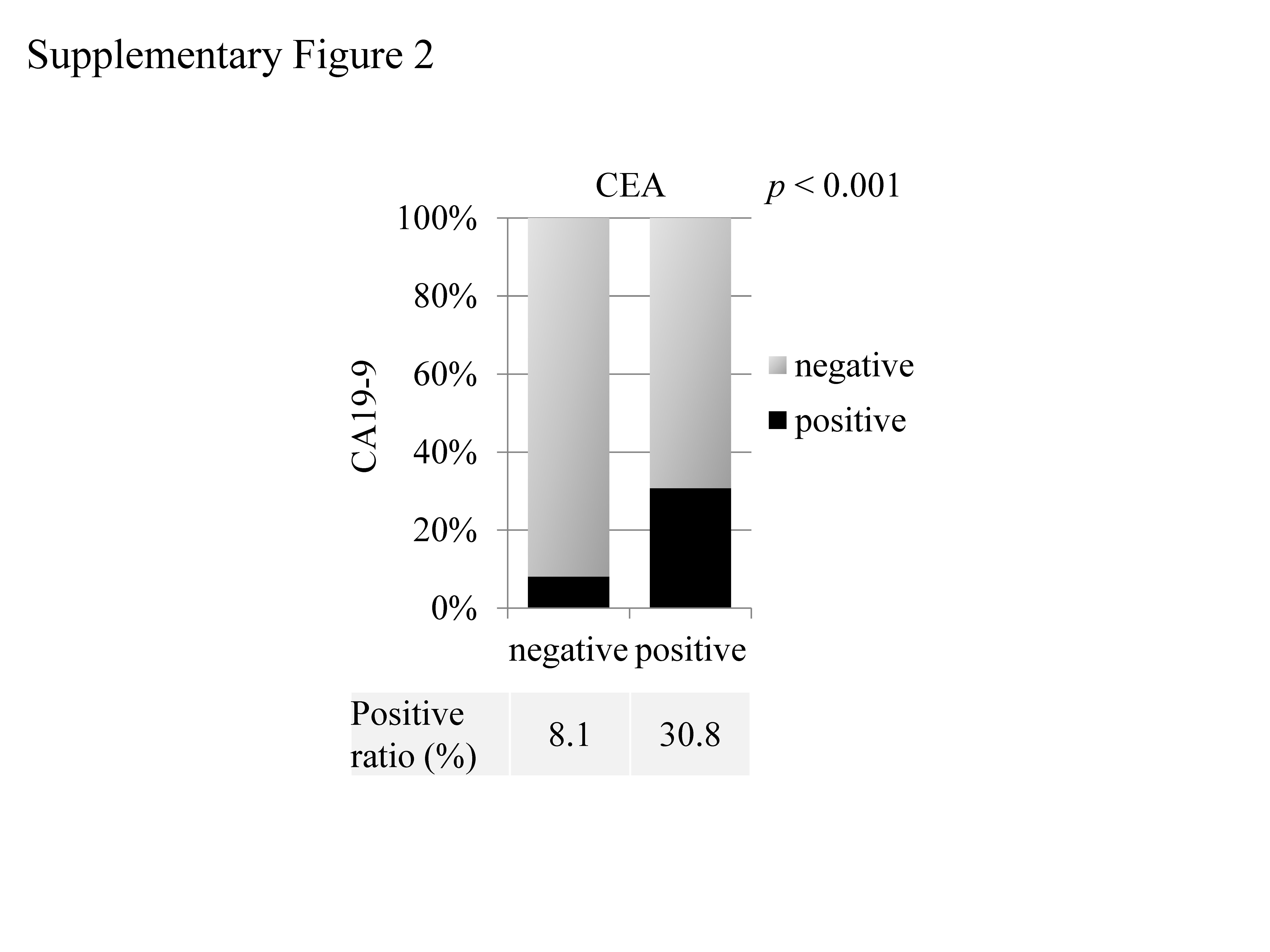

Supplement: Supplementary file 2 [file cam40004-1659-sd2.tiff]
