# Supplementary material for: Carbohydrate antigen 19-9 is a useful prognostic marker in esophagogastric junction adenocarcinoma
Source: Cancer Med. 2015 Aug 26;4(11):1659–66. doi: 10.1002/cam4.514 (PMC4673992; doi:10.1002/cam4.514)
Supplement: Supplementary file 5 [file cam40004-1659-sd5.docx]

**SUPPLEMENTARY FIGURE LEGENDS**

**SUPPLEMENTARY FIG. 1** Flow diagram for included cases for this study

*CEA* carcinoembryonic antigen, *CA19-9* carbohydrate antigen 19-9

**SUPPLEMENTARY FIG. 2** Associations between preoperative serum CEA and CA19-9.

*CEA* carcinoembryonic antigen, *CA19-9* carbohydrate antigen 19-9

**SUPPLEMENTARY FIG. 3** (a), (c), (e): Cancer-specific survival curves according to preoperative CA19-9 positivity in T1, T2-3, or T4 tumors. (b), (d), (f): Overall survival curves according to preoperative CA19-9 positivity in T1, T2-3, or T4 tumors.

*CA19-9* carbohydrate antigen 19-9

**SUPPLEMENTARY FIG. 4** (a) Cancer-specific and (b) overall survival curves in combination of CEA and CA19-9 positivity status.

*CEA* carcinoembryonic antigen, *CA19-9* carbohydrate antigen 19-9
